# The Multiple Facets of ATRX Protein

**DOI:** 10.3390/cancers13092211

**Published:** 2021-05-05

**Authors:** Martina Valenzuela, Roberta Amato, Antonella Sgura, Antonio Antoccia, Francesco Berardinelli

**Affiliations:** 1 Department of Science, University Roma Tre, 00146 Rome, Italy; martina.valenzuela@uniroma3.it (M.V.); roberta.amato@uniroma3.it (R.A.); antonella.sgura@uniroma3.it (A.S.); antonio.antoccia@uniroma3.it (A.A.); 2 Laboratory of Neurodevelopment, Neurogenetics and Molecular Neurobiology Unit, IRCCS Santa Lucia Foundation, 00143 Rome, Italy

**Keywords:** ATRX, Epigenetics, chromatin remodeling, telomere, replicative stress response, genome stability

## Abstract

**Simple Summary:**

The gene encoding for the epigenetic regulator ATRX is gaining a prominent position among the most important oncosuppressive genes of the human genome. ATRX gene somatic mutations are found across a number of diverse cancer types, suggesting its relevance in tumor induction and progression. In the present review, the multiple activities of ATRX protein are described in the light of the most recent literature available highlighting its multifaceted role in the caretaking of the human genome.

**Abstract:**

ATRX gene codifies for a protein member of the SWI-SNF family and was cloned for the first time over 25 years ago as the gene responsible for a rare developmental disorder characterized by α-thalassemia and intellectual disability called Alpha Thalassemia/mental Retardation syndrome X-linked (ATRX) syndrome. Since its discovery as a helicase involved in alpha-globin gene transcriptional regulation, our understanding of the multiple roles played by the ATRX protein increased continuously, leading to the recognition of this multifaceted protein as a central “caretaker” of the human genome involved in cancer suppression. In this review, we report recent advances in the comprehension of the ATRX manifold functions that encompass heterochromatin epigenetic regulation and maintenance, telomere function, replicative stress response, genome stability, and the suppression of endogenous transposable elements and exogenous viral genomes.

## 1. Introduction

Alpha Thalassemia/mental Retardation syndrome X-linked (ATRX) is a member of the switch/sucrose non-fermentable (SWI-SNF) protein family [1,2,3]. SWI-SNF proteins play roles in several biological processes, such as DNA recombination and repair, transcriptional regulation, and remodeling of nucleosomes [2]. In the last years, accumulating evidence point to the prominence of chromatin regulation in processes such as development and cancer. To this end, ATRX represents a paradigmatic example showing as mutations in the same gene can cause both syndromic intellectual disability or cancer, depending on the timing and the type of mutation [4].

**Figure 1:**
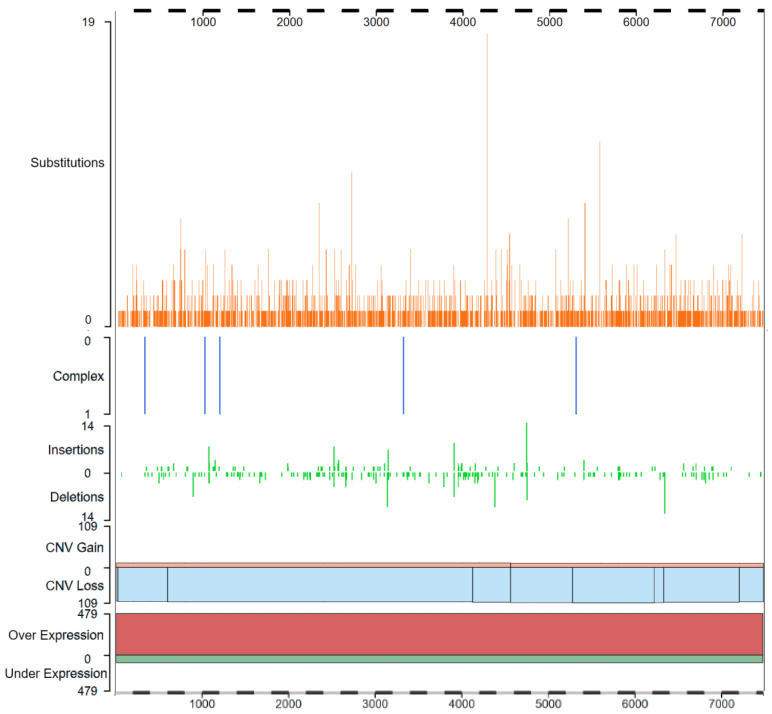
ATRX mutations in cancer—The gene histogram shows the distribution of all mutations contained across the ATRX gene in 3357 cancer samples and cataloged in the COSMIC database (v92; GRCh38) https://cancer.sanger.ac.uk/cosmic/gene/analysis?coords=bp%3AAA&wgs=off&id=230763&ln=ATRX&start=1&end=2493 (accessed on 16 April 2021). From top to bottom are shown single nucleotide substitutions (orange), multinucleotide substitutions (“complex”) (blue), insertions and deletions (green), copy number gain (pink)/loss (blue), gene over (red)/under (green) expression. Gene overexpression was found in 479 samples whereas underexpression was found in 102 samples. Image modified from COSMIC database website [15,16].

Indeed, hypomorphic germ line mutations of human ATRX gene give rise to the ATRX syndrome, a disorder characterized by a form of X-linked severe intellectual disability, facial dysmorphism, reduced expression of the α-globin genes (α-thalassemia), urogenital dysfunction, and skeletal abnormalities [5]. On the contrary, de novo null mutations in the ATRX gene have been detected in at least 15 different types of cancers including neuroblastoma, glioma, pancreatic neuroendocrine tumors (panNETs), and pediatric osteosarcoma [6,7,8,9,10,11,12,13] and recently ATRX gene status has become a critical marker that defines molecular World Health Organization (WHO) classification of tumors of the central nervous system, such as glioma [14]. A gene histogram obtained from the Catalogue of Somatic Mutation in Cancer (COSMIC) database (https://cancer.sanger.ac.uk/cosmic (accessed on 16 April 2021)) showing somatic mutations of the ATRX gene identified on 3357 cancer samples were reported in Figure 1.

Such severe effects of mutations in the ATRX gene entail a fundamental role in normal cellular function that justify the increasing interest of the scientific community on this major tumor suppressor gene. In this review, we provide an updated overview of the multiple functions of the ATRX protein. In particular, the role played by ATRX in pericentromeric and telomeric heterochromatin formation and maintenance, endogenous and exogenous viral genome heterochromatinization, X chromosome inactivation (XCI), and replicative stress (RS) response (RSR) will be discussed. 

## 2. ATRX Gene and Protein

In humans, the ATRX gene lies on the long arm of the X chromosome in position 21.1. It spans about 300 kb of genomic DNA and is composed of 36 exons [2]. It is highly conserved between mouse and human (85% homology) and protein homologs have been identified in Drosophila melanogaster (dATRX; 66% homology) and Caenorhabditis elegans (XNP-1; 52% homologous) [2,17,18]. ATRX is a ubiquitously expressed nuclear protein with highest levels in fetal brain, suggesting an important role in brain development [19]. The complete cDNA for the ATRX gene extends for 11.165 nucleotides, but there are several alternatively spliced transcripts that encode for proteins with different molecular weights [2,20]. Depending on the presence or absence of exon 6 the gene gives rise to proteins of 282 or 278 kDa, respectively [20], and this alternative splicing has also been conserved in mice which encodes for proteins of 279 or 274 kDa [21]. 

**Figure 2:**
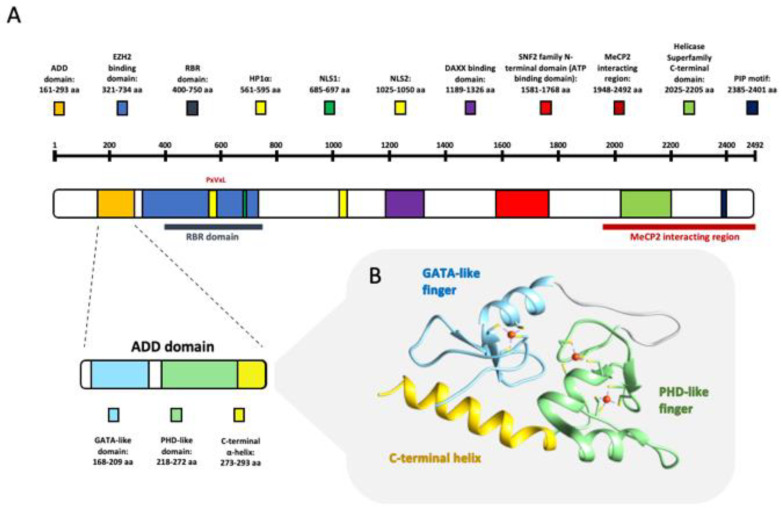
ATRX protein. (**A**) Domain organization of the ATRX protein are depicted using different colors. Localization and aminoacids involved in each domain are reported in the figure key. ADD domain, the most characterized region of the protein is enlarged to show GATA-like, PHD-like and C-terminal subdomains [3,26,27,28]. (**B**) Three-dimensional structure of the ADD domain obtained through UCF Chimera Software is represented.

The inclusion of exons 7 in the transcript introduces in-frame stop codon giving a predicted protein of 260 kDa [2] while the inclusion of intron 34 results in a truncated protein of 267 or 265 in human and mouse, respectively [21]. In addition to these variants, another isoform, with a smaller molecular mass (~200 kDa) was also observed [22]. This truncated isoform, called ATRXt, originates from the non-removal of the intron 11 of the transcript and has been highly conserved between mouse and human [22]. The resulting truncated protein colocalizes with full length ATRX at blocks of pericentromeric heterochromatin (PCH) but is not present at promyelocytic leukemia protein (PML) nuclear bodies (PML-NBs) (ATRX localization at PML-NBs will be discussed in Section 3). Its biological function has not been fully clarified but its ability to interact with full length ATRX protein [22] suggests that it may have a role in regulating its activity in heterochromatic regions [23]. Looking at the structure of the ATRX protein three main regions can be distinguished: a helicase/ATPase C-terminal domain that provides DNA-dependent ATPase activity and identifies ATRX as a member of the SNF2 family of chromatin-associated proteins; a centrally located DAXX-binding motif [24,25] alternating hydrophilic and hydrophobic regions; a N-terminal hydrophilic region that contains a nuclear localization signal (NLS) (Figure 2) [2].

The N-terminal domain is, in turn, divided into three modules. Starting from the N-terminus, there are two different types of zinc-finger motifs: the first one binds a single zinc ion through four cysteines (C2C2 motif) and is structurally very similar to the zinc fingers of the erythroid transcription factor GATA-1, suggesting that this domain plays a role in DNA binding [3,29]. The second one binds two zinc ions and shares homology with the plant homeodomain (PHD; Cys4-His-Cys3) characterized for the first time in Arabidopsis Thaliana [30]. The latter domain has been identified in more than 100 proteins, many of which are implicated in chromatin-mediated transcriptional control [31]. This peculiar domain organization is shared only with methyltransferases DNMT3A, DNMT3B, and DNMT3L [32] and has been named the ATRX-DNMT3-DNMT3L (ADD) domain [33]. The last part of this N-terminal domain is a 25 aa long C-terminal α-helix (Figure 2) that interacts with the GATA 1 N-terminal finger bringing the N and C termini of ADD domain close together to form a single globular domain [3]. The mutations that cause the ATRX syndrome are often located within the putative ATPase/helicase domain and the PHD motif of ADD domain [2,5,34], whereas tumorigenic mutations of ATRX are randomly localized point mutations, determining protein loss of function (Figure 1) [7,35,36]. Table 1 shows an updated list of ATRX direct interactors, ATRX domain involved in the interactions, and the cellular process in which the complex takes part.

## 3. ATRX Interacts with DAXX in PML Nuclear Bodies Promoting H3.3 Deposition

ATRX achieves its main role when interacts and binds the chaperone protein DAXX, forming a chromatin remodeling complex involved in replication independent histone variant H3.3 deposition and incorporation at PCH, telomeres, and in others transcriptionally silent genomic regions known to be enriched with H3K9me3, H4K20me3, and DNA methylation [24,37,55,56,57]. 

The canonical form of histone H3 is represented by two proteins, H3.1 and H3.2, which differ by only one amino acid and are both encoded by multiple genes. H3.1 and H3.2 are deposited at replication forks by the chromatin assembly factor 1 (CAF1) complex in a S-phase dependent process [58,59,60]. Histone H3.3 is a H3 variant expressed throughout the cell cycle and encoded by two genes (H3F3A and H3F3B). It differs from the canonical H3.1 and H3.2 in four and five amino acids, respectively. These differences enable the H3.3 to interact with the ATRX/DAXX complex and allow nucleosomes assembly also in interphase [55,61,62]. Both ATRX and DAXX seem to be equally important in this process: DAXX provides the H3.3 binding specificity and chaperone activity while ATRX targets DAXX-dependent H3.3 deposition at H3K9me3-enriched chromatin through the ADD domain [51,63]. ChIP-seq of H3.3 in WT and ATRX KO mouse ESCs showed that disruption of the ATRX/DAXX complex determined H3K9me3 loss, thus proposing H3.3 deposition as a central process in H3K9me3 constitutive heterochromatin maintenance and in epigenetic memory of silenced heterochromatin [57]. A role in chromosome segregation has also been observed in mouse oocytes at the metaphase II stage, indicating ATRX is required for centromere stability and the epigenetic control of heterochromatin function during meiosis [64].

**Figure 3:**
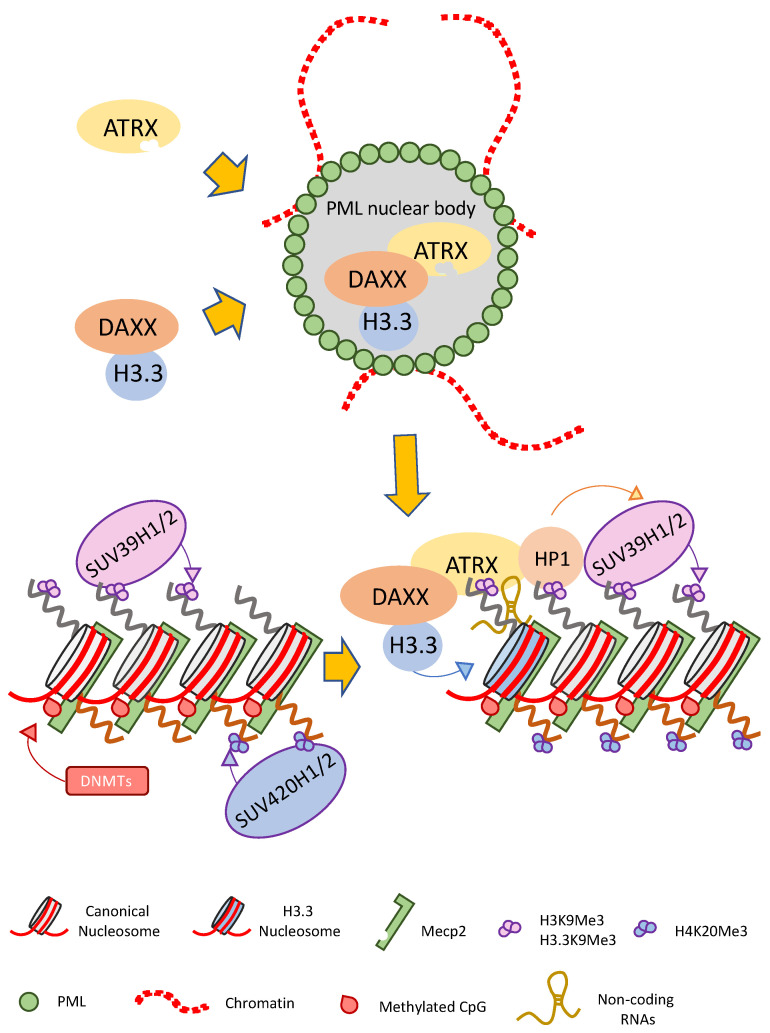
Pericentromeric chromatin (PCH) establishment and maintenance is mediated by the ATRX-DAXX complex. DAXX binds the H3.3 and locates it to PML-NBs where the complex is assembled. The complex recognizes H3K9Me3 and promotes H3.3 deposition at PCH. Several other proteins and non-coding RNAs are involved in the process activating a “self-sustaining loop” mechanism.

Histone variant H3.3 is found in both transcribed genes and in pericentromeric and telomeric constitutive heterochromatin [55,65] but its deposition in different sites is mediated by different mechanisms. ATRX/DAXX complex plays a role in the deposition of histone variant H3.3 at pericentromeres and telomeres and other repetitive elements, but not in transcribed genes [55,61,62] where it is instead deposited by a different chromatin remodeling complex, named HIRA complex [66,67]. Evidence from the literature suggest that DAXX also has a role in regulating the cellular localization of ATRX protein through its recruitment to PML-NBs (Figure 3) [24,37].

PML-NBs consist of dynamic foci composed by a shell of PML permanently or transiently associated with more than 100 other proteins [68,69,70] including newly synthesized H3.3 [71] and the ATRX/DAXX complex [72]. PML-NBs are involved in the regulation of many cellular functions, such as the cell cycle, apoptosis, response to virus infections, gene expression, stress, senescence and DNA damage responses [73,74,75,76,77]. The presence of the ATRX/DAXX/H3.3 complex at PML-NBs suggests that inside these bodies H3.3 interacts with the complex to be subsequently deposited on specific chromatin sites [71]. Indeed, PML can be found in heterochromatic regions such as telomeres [78,79] or other extensive heterochromatic regions where it favors the deposition of H3.3 by ATRX. Loss of PML impairs deposition of H3.3 by ATRX and DAXX in these sites, demonstrating a PML-dependent role of ATRX/DAXX in the deposition of H3.3 [80]. Moreover, this loss leads to a shift in H3 methylation patterns, from H3K9me3 to H3K27me3, a well-known repressive epigenetic mark present in facultative heterochromatin installed by a complex of four proteins (EZH1/2, SUZ12, EED, and RbAp46/48) known as Polycomb Repressive Complex 2 (PRC2). This could be explained by the ability of ATRX to bind and recruit PRC2 independently from PML and point to an alternative way to preserve heterochromatin when the PML-NBs are absent and the H3.3 deposition by ATRX/DAXX is halted [48,80].

Interestingly, ATRX depletion in fibroblasts leads to a shift in PRC2 localization and function, with a redistribution to ectopic sites and within coding genes, causing a misdeposition of the H3K27me3 in these sites [48]. For instance, it was recently showed that ATRX knockout in GBM cells triggers [81] an impoverishment of H3K27me3 in FADD (Fas-associated death domain) promoter region, suggesting that the ATRX/EZH2 complex promoted trimethylation of histone H3K27 in this region, thus silencing it [82]. In addition, loss of ATRX in malignant peripheral nerve sheath tumors led to an upregulation of PRC2 gene targets expression, thus giving another prove that ATRX deficiency causes a re-expression of silenced genes controlled by PRC2 [48,83].

## 4. ATRX Contributes to Pericentromeric and Telomeric Heterochromatin Formation and Maintenance

In the absence of ATRX, chromatin accessibility (in both repetitive and non-repetitive DNA) undergoes significant changes leading to transcription of regions normally suppressed [81,84]. Among these regions are included also constitutive heterochromatic regions such as PCH and telomeres [85,86,87].

### 4.1. ATRX Role at Pericentromeric Heterochromatin

PCH is a particular form of constitutive heterochromatin that is localized to both sides of centromeres and that forms silent compartments enriched in repressive marks. PCH organization and maintenance of its repressive state is tightly regulated by a plethora of factors, including enzymes (e.g., DNA methyltransferases—DNMTs, histone deacetylases—HDACs, and histone methyltransferases—HMTs), DNA and histone methylation binding factors (e.g., MECP2 and HP1), chromatin remodeling proteins (e.g., ATRX), and non-coding RNA (ncRNA) (Figure 3). Histone tails post-translational modifications are crucial events for the preservation of PCH molecular organization and are mediated by the concerted actions of a number of enzymes [88]. In particular, trimethylation of H3K9 is mediated by the histone methyltransferases (HMT) suppressor of variegation 3–9 homolog 1 and 2 (SUV39H1/2) [89,90] and partially depend on DNA methylation [91]. Proteins belonging to the heterochromatin protein 1 (HP1) family (HP1s, i.e., HP1α, β and γ) are involved in PCH formation, preservation and propagation [92,93]. The process depends on HP1s binding to H3K9me3 installed by SUV39H1/2 and is reinforced by a “self-sustaining loop” mechanism in which HP1s, in turn, recruit the SUV39H1/2 [91,94,95,96,97]. Interestingly, the knockout of Suv39H1 and Suv39H2 abrogates ATRX presence at PCH. In turn, the absence of ATRX significantly decreased HP1α and HP1γ enrichment at PCH, supporting the hypothesis that ATRX is important for HP1s recruitment [98]. ATRX associates with HP1 [63,65,99] via the conserved pentapeptide HP1-interacting motif Pro-Xaa-Val-Xaa-Leu (PxVxL) [51,100], that allows ATRX and HP1 colocalization within PCH [39,101].

In addition, it has been shown that the ATRX directly interacts with Methyl-CpG binding protein 2 (MeCP2), an epigenetic reader belonging to the methyl-binding domain (MBD) family. ATRX interacts with MeCP2 through its C-terminal helicase motif and loss of MeCP2 disrupts ATRX localization at PCH (Figure 3) [40]. ATRX and MeCP2 interaction has also been implicated in the expression of a network of imprinted genes in the postnatal mouse brain, such as H19/Igf2 and Gtl2/Dlk1 [53]. Furthermore, ATRX and MeCP2 are reciprocally dependent both for their expression and targeting to PCH [98]. Recently it has been suggested that ATRX localization to PCH is also mediated by binding of specific ncRNAs. Indeed, immunofluorescence and ChIP analysis showed that MeCP2 deficient murine embryonic stem cells displayed a decrease of ATRX enrichment at PCH following RNase A treatment [98]. Indeed, ATRX is an avid and specific RNA binding protein as shown by its interaction with different RNAs such as the telomeric repeat containing RNA (TERRA) (see Section 4.2 and Section 5), the X-inactive specific transcript (Xist) RNA (see Section 6) and with the muscle-specific Chromatin reorganization 1 (ChRO1) ncRNA in myotubes [49]. Very recently, a putative RNA binding region (RBR) was identified in the N-terminal of the protein and deletion of RBR determine a diminished localization to chromatin and modify its enrichment in specific sites (Figure 2) [27].

### 4.2. ATRX Role in Telomeric Heterochromatin

Telomeres are nucleoprotein structures that cap the ends of eukaryotic chromosomes consisting of tandem G-rich repeats (TTAGGG)n and playing a central role in genome stability. In mammalian cells, telomeric chromatin contains epigenetic markers characteristic of silenced chromatin such as those found at PCH [102,103,104]. In particular, telomeric chromatin is enriched in H3K9me3, H4K20me3, and is associated to HP1α (and its homologous in mice, CBX5) [105]. DNA methylation of subtelomeric repeats reinforces this heterochromatic state. However, the evidence supporting the heterochromatic state of telomeres has been collected mostly in murine models [106], whereas in humans the epigenetic nature of telomeres is still debated [107,108] and a number of works showed unexpected low levels of H3K9me3 (and high levels of H3K27Ac) in a panel of human cell lines (normal and tumoral), challenging the heterochromatic state of human telomeres [109,110,111,112]. Interestingly, clear heterochromatic marks such as H3K9me3 and DNA hypermethylation characterize instead human subtelomeric regions [110,111]. Despite this uncertainty, it is widely accepted that telomeric heterochromatin is important for preventing the recruitment to telomeres of DNA damage-signaling proteins and thus avoiding homologous recombination events that use telomeric DNA as a template [87]. Remarkably, the deletion of factors involved in heterochromatin assembly, such as (i) SUV39H1/H2 and SUV420H1/H2, which catalyze H3K9me3 and H4K20me3 and (ii) DNA methyltransferases (DNMT 3a/b 1), which methylate subtelomeric regions, has been found to cause abnormal telomeric recombination and lengthening [102,104,105,113,114].

**Figure 4:**
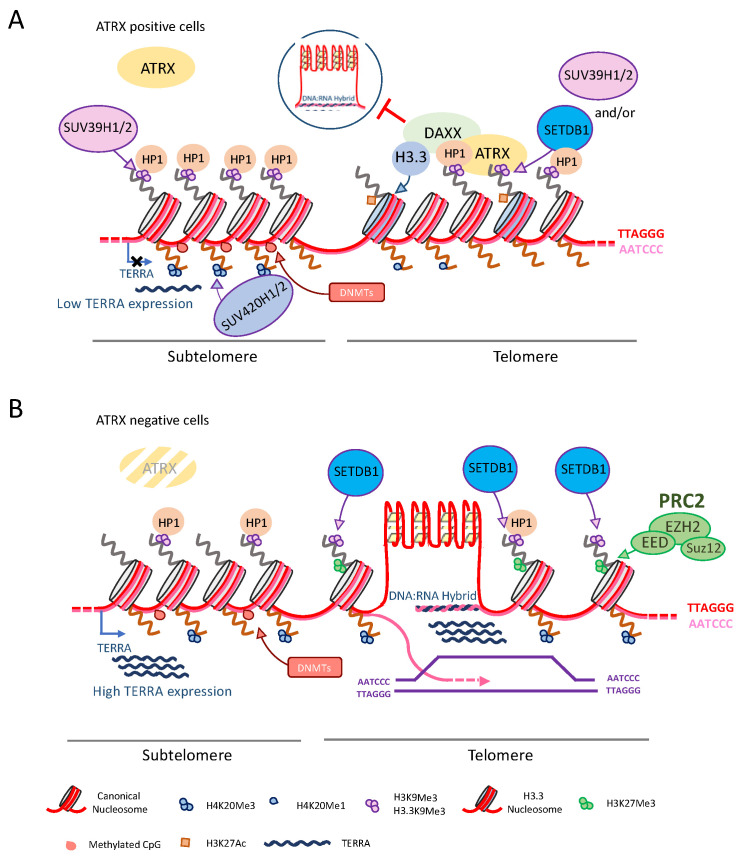
(**A**) ATRX role in the maintenance of telomeric and subtelomeric chromatin. ATRX localization at telomeres is independent from SUV39H1/2 and probably is determined by SETDB1 H3K9Me3 deposition and by the action of TERRA. (**B**) In the absence of ATRX, TERRA expression strongly increases and DNA:RNA hybrids (R-loops) occur at telomeres hindering telomere replication and favoring the onset of recombination mediated processes at telomeres that characterize ALT cells.

In addition, the depletion of ATRX triggers a progressive reduction of telomere nucleosome density, a feature of cancer cells exploiting the alternative lengthening of telomere (ALT) telomere maintenance mechanism (TMM) [115,116]. As previously discussed for pericentromeric heterochromatin, PRC2 can mediate H3K27me3 also at telomeres and it was recently showed that this process is dependent by the telomeric transcript named Telomeric Repeat containing RNA (TERRA) and positively affects, H3K9me3, H4K20me3, and HP1 deposition in the absence of ATRX [117] (Figure 4).

Whereas ATRX localization to pericentromeric heterochromatin is dependent on H3K9me3, it is not clear if the same is true for telomeric heterochromatin. Indeed, abolition of H3K9me3, through the inactivation of the histone methyltransferases SUV39H1/2, determines an increase of ATRX binding at telomeres [118]. This evidence suggests that ATRX is recruited to GC-rich tandem repeats through a H3K9me3-independent mechanism, probably involving TERRA. Another possibility that was recently proposed is that H3K9me3 deposition at telomeres is not SUV39H1/2-dependent but mediated by the SET Domain Bifurcated 1 (SETDB1) histone methyltransferase. The authors showed that the removal of SUV39H1/2 led to H3K9Me3 impoverishment at pericentromeres and HP1 relocation from pericentromeres to telomeres, thus stimulating SETDB1-dependent H3K9me3 deposition and ATRX binding [106].

## 5. ATRX Participates in DNA Repair Process

### 5.1. ATRX Prevents Replication Stress in Specific Regions of the Genome

There is recent evidence showing that ATRX has a role in the maintenance of genomic stability and may participate in replication stress prevention [44,119]. The ways in which this process takes place are not yet fully understood. Recently, it was shown that ATRX is a novel physical and functional interactor of FANCD2, acting during early steps of HR-associated DNA repair. In this regard, ATRX/DAXX complex interacts with the FANCD2/MRN complex reinforcing their interaction and creating a “super-complex” [41]. It was already known that ATRX associates with the MRE11-RAD50-NBS1 (MRN) complex during the S phase and this interaction is important in DNA replication and in genomic stability maintenance [42,43,119]. On the other hand, FANCD2 has been found to play a role in replication stress response protecting stalled replication forks from nucleolytic degradation, promoting their restart and simultaneously suppressing the firing of new replication origins [41,120,121]. In order to stimulate HR-mediated replication fork recovery and DSB repair, the ATRX/DAXX-MRN-FANCD2 supercomplex assembles on chromatin and promotes the deposition of histone variants H3.1 and H3.3 and recruits HR and replication restart key factors such as RAD51 [42,122] and CtIP [41]. A recently published paper suggests that ATRX (together with DAXX) takes part in DNA two-ended Double-Strand Break (DSB) repair inhibiting RECQ5 mediated Synthesis-Dependent Strand Annealing (SDSA) and promoting a different HR subpathway [44,123]. This process involves extended DNA repair synthesis, D-loop formation, the creation of dHJ, and their resolution by the nuclease Mus81 potentially generating Sister Chromatid Exchanges (SCEs) [124,125]. In this model, ATRX performs its function at later stages of HR, after RAD51 removal by RAD54 [44] competing with RECQ5 and determining the HR subpathway [123]. DNA repair synthesis progression is closely coupled with incorporation of H3.3 by ATRX/DAXX complex, which is essential to overcome topological constraints at the moving D-loop. The association between the DNA repair and chromatin reorganization is supported by the interaction between RFC-1, PCNA and ATRX that interact through PIP consensus sequence (Figure 2). In the absence of ATRX, HR occurs through RECQ5-dependent SDSA in a Mus81-independent way. The latter pathway does not entail the formation of dHJ and crossover products. These evidence are contradictory with the well accepted role of ATRX in the suppression of HR in telomeric regions suggesting that the regulation of recombination sub-pathways might be fundamentally different in genomic and telomeric regions [44,123].

Overall, the emerging scenario suggests that ATRX can act at several steps during HR repair, similar to other HR factors such as BRCA1 or members of the RAD51 paralog family [126,127], and its role may be dependent on the cell cycle stage and/or the type of DNA lesion [41]. In line with the prevention of RS, ATRX plays a role in specific genome sites, in particular it binds G-rich sequences which tend to assume non-B conformations under physiological conditions, including G-quadruplexes [56]. These structures tend to form in single stranded DNA (ssDNA) for instance at telomeric G-rich overhang or in genomic DNA during transcription and replication [56]. Indeed, a lot of evidence indicates that these secondary structures become an obstacle for replication fork progression, leading to fork stalling or collapse [119,128,129]. ATRX seems to act indirectly on these structures, as it does not seem to possess G-quadruplex unwinding activity itself [119]. Indeed, ATRX in complex with DAXX, is able to prevent the formation of these structures through the deposition of histone H3.3, facilitating replication [130,131] and the passage of RNA polymerase [4,132].

### 5.2. ATRX Suppress Homologous Recombination at Telomeres

Certain regions of the human genome, such as common fragile sites and telomeres, are particularly sensitive to DNA replication stress due to their inherently ‘difficult-to-replicate’ nature [133]. Both the replication fork collapse and the bypass of replication-blocking lesions generate single strand breaks (SSB) and DSB that are normally repaired by HR pathways. However, due to their nature of tandemly repeated sequences, telomeres need to strictly repress HR and all the manifold roles played by ATRX at telomeres seem to guarantee the suppression of HR. It has been proposed that the absence of ATRX promotes the transcription of TERRA, inducing an increase of non-canonical secondary structures at telomeres such R-loops and G4. Indeed, R-loops are DNA:RNA hybrids formed during the transcription of G-rich repetitive DNA sequences and involve principally the DNA C-rich strand [134]. This implies the stabilization of the G-rich strand, favoring the formation of G4 structures [135] (Figure 4). The presence of R-loops and G4 DNA has both been implicated in stalling of replication, causing a subsequent DNA damage response [118,129,136,137] and prompting recombination at telomeres [138,139]. In favor of this evidence, it appears that stabilization of these structures via G4 ligands increases HR at telomeres [140,141] and decreases the ability of ATRX to suppress ALT phenotype [139].

The ATRX-dependent cell cycle regulation of TERRA and Replication Protein A (RPA) removal from telomeres following replication [86] are two events closely related to each other and support the role of ATRX in suppressing the ALT pathway. Indeed, RPA transiently associates with ssDNA during S phase and recruits ATR, a protein kinase that is a key regulator of HR [86,142]. The association of RPA with telomeres in S phase is facilitated by TERRA and its release in G2/M phase coincides with the repression of TERRA by ATRX [143] consequently, loss of ATRX results in a persistent association of the RPA protein with telomeres [86]. Moreover, ALT activation appears to depend on ATR-dependent sensing of RS [139,144]. These two pieces of evidence corroborate the notion that ATRX is involved in the suppression of telomeric recombination and ALT pathway [139]. In addition, ATRX is thought to inhibit ALT also by sequestering the MRN complex away from PML [139]. The MRN complex, catalyzes the 5′ to 3′ resection of the DNA ends and promotes subsequent strand invasion facilitating HR. Its association with PML bodies appears to be needful to the ALT pathway activation and likely constitutes the sites of telomeric recombination [139,145]. A further role that ATRX plays to telomeres is to participate in the resolution of telomeric cohesion before entering in mitosis. Cohesion between sister chromatids is important as it provides a template for recombination and repair during and after DNA replication in S and G2 phases of the cell cycle [146]. The main mediators of telomeric cohesion are the cohesin subunit SA1 and the two shelterin proteins TRF1 and TIN2 [147,148,149] whereas telomeres cohesion resolution requires the TRF1-binding PARP and telomeric poly(ADP-ribose) tankyrase 1 [150]. Tankyrase 1 is an enzyme found at telomeres in late G2/early mitosis and it PARylates itself and TRF1 [151]. ATRX has no direct role in resolving telomeric cohesion, but interacts indirectly with Tankyrase 1, through the link with the histone macroH2A1.1 variant which is the only one of the three variants (macroH2A1.1, 1.2, and 2) able of binding PAR [46,152,153]. In the absence of ATRX, macroH2A1.1 is free to sequester Tankyrasi 1 away from telomeres preventing it from playing its role in resolving cohesion [46]. The authors reported that the persistent telomere cohesion in mitosis promotes sister telomere recombination in ALT cells [46]. On the contrary, a recent study demonstrated that the loss of ATRX caused a telomere-specific cohesion defect in postmitotic interphase that enables interactions between nonallelic telomeres favoring out-of-register telomere recombination and thus telomere elongation [154]. ATRX is also able to influence HR by modulating the deposition of alternative macroH2A1 splicing isoforms named macroH2A1.2 [155] involved in the RS-dependent recruitment of tumor suppressor BRCA1 [156]. BRCA1 plays a role in both DSB- and stalled replication forks-repair through HR as well as break-induced replication (BIR) [157,158]. BIR is a process that has been found orchestrating homology-directed telomere maintenance in ALT tumors, consisting in conservative DNA synthesis upon DNA break formation and subsequent strand invasion [157,158]. Telomeres are particularly enriched with macroH2A1.2, especially in ALT cells. The loss of ATRX leads to a transient decrease in macroH2A1.2 during acute RS, which causes an increase in DSB formation and prompt ALT initiation. MacroH2A1.2 is then redeposited at DSB sites, facilitating homology-directed repair and promoting ALT [155].

## 6. ATRX Role in X Chromosome Inactivation

ATRX has been also found to colocalize with heterochromatin on the inactivated X (Xi) chromosome [47,159] and, as discussed for facultative heterochromatin (see Section 3 and Section 4), also in this case is essential for PRC2 localization [48]. In particular, PRC2 methyltransferase activity is critical in X-chromosome inactivation (XCI), mediated by the noncoding RNA XIST, which is transcribed only from the Xi and acts in cis along the chromosome [160,161]. ATRX binds XIST and interacting with the EZH2 subunit, drives the PRC2 complex on the Xi chromosome, in the XIST targeted zones [48]. Indeed, the loss of ATRX deregulates PRC2 ability to tri-methylate histone H3 at lysine 27 (H3K27) preventing the establishment of repressive chromatin [48,162,163].

ATRX has been shown to interact with another important participant to XCI, the histone variant macroH2A. This histone variant was first detected to be enriched in the inactive X of female mammals [164], and its association to the inactive X takes place after the initiation and propagation of X-inactivation [165]. Its role seems to be redundant, in fact knockdown and knockout of both histone isoforms, macroH2A1 and macroH2A2, in mouse does not affect initiation and maintenance of XCI [166,167,168]. In mouse embryonic fibroblasts (MEFs), ATRX and macroH2A were found to colocalize at the Xi by immuno-FISH analysis [47]. This interaction can be supported by the fact that macroH2A is known to interact also with EZH2 [169], and large macroH2A-bound regions overlap with H3K27me3 mark [170]. It is thus conceivable that ATRX may function as a platform that links PRC2 complex and macroH2A histone by directing them on the Xi, enforcing its silencing. It is worth to note that ATRX gene expression is not suppressed in X chromosome undergoing inactivation, hence in females its expression remains biallelic. This makes ATRX an “escape” gene and biallelic expression of ATRX (and other 5 X-linked tumor suppressor genes) strongly associates with the lower incidence of a number of human cancers such as lower-grade gliomas in female individuals [171].

## 7. ATRX Is Involved in Repression of Transposon, Retrotransposon and Viral DNA 

Importantly, ATRX is involved in the repression of endogenous retroviral elements. In mouse ESCs ATRX and DAXX deletion resulted in a decrease of H3.3 enrichment and H3K9me3 at class I and class II ERVs (endogenous retroviral elements), and in particular those of the early transposon (ETn)/MusD family and intracisternal A-type particles (IAPs), demonstrating the crucial role of ATRX-DAXX complex in the regulation and silencing of endogenous retroviral element [172]. ChIP-seq analysis demonstrated that both DAXX and ATRX co-occupied class I and II ERVs. In these sites, H3.3 deposition by ATRX-DAXX complex was mediated by KAP1, also known as TRIM28 [173], as demonstrated by the reduction of H3.3 in these sites when KAP1 was depleted [172]. This process stimulates a positive feedback (since KAP1 is recruited to H3.3-containing chromatin) and maintains a silenced state at ERVs in collaboration with H3K9me3 by the ESET complex [172]. These results were confirmed in another study on mouse ESC where KAP1 knockdown in Atrx ko cells led to a severe derepression of IAP retrotransposons and enhanced heterochromatin accessibility in contrast with WT cells [174].

Furthermore, ATAC-seq analysis in a human liver cancer cell line knocked out for ATRX identified 7 retrotransposons and 43 long terminal repeats (LTR) where heterochromatin accessibility was increased [175]. Taken together, these data indicate the importance of ATRX in avoiding aberrant transposon activation leading to illegitimate homologous recombination, chromosome breaks and translocations [176] and highlight the critical role of ATRX in silencing repetitive elements through regulation of H3.3 and H3K9me3 deposition.

The PML-NBs/ATRX-DAXX axis is also one of the first players in the defense against viral infection, through direct association and silencing [74,177]. Knockdown of constitutive PML NB proteins, such as PML, SP100, ATRX or DAXX, leads to human cells inability to suppress Herpes Simplex Virus 1 (HSV-1) replication [178,179,180,181]. In PML KO mice and in PML stably silenced BJ cells infected by latent HSV-1 have reported an impairment of ATRX and DAXX colocalization with the viral latent genome [182]. Furthermore, the same study showed that latent/quiescent viral genomes were almost exclusively chromatinized with H3.3 and its methylated form H3.3K9me3, and ATRX/DAXX and HIRA complexes were found to interact with various viral loci, suggesting that this chromatinization can be triggered by ATRX/DAXX complex in association with the PML-NBs [182]. In fibroblasts infected by HSV-1, ATRX, and PML recruitment to viral DNA was detected by 15 min post infection, and ATRX recruitment at HSV-1 DNA was shown to be DAXX-dependent. Although ATRX was demonstrated to inhibit viral mRNA expression, it had no effect on the initial heterochromatinization of virus genome at early times post infection. However, it was required for the maintenance of viral heterochromatin during chromatin stress, such as replication and transcription [183]. In addition, ATRX/DAXX complex also appears to be important for maintaining DNA virus latency and a depletion of ATRX or DAXX was able to induce Epstein–Barr Virus (EBV) reactivation from latently infected cells [184]. It is not surprising that HSVs has evolved strategies to disrupt PML- NBs, and, as reported by Jurak and colleagues in 2012, to inhibit ATRX by the use of miRNAs (miR-H1 and miR-H6 in HSV-1 and HSV-2, respectively). In addition, the HSV intermediate early protein ICP0 is involved in the reduction of ATRX protein levels, and the tegument protein, VHS, is required for depletion of ATRX mRNA, indicating that HSV is equipped with multiple mechanisms to limit the expression of ATRX and alleviate its repression of viral gene expression [185].

## 8. Concluding Remarks

Given the manifold roles played by ATRX in the regulation of chromatin compaction, in the response to replicative hindrances and in the maintenance of genome stability and telomere function, we believe that the contribution of ATRX to critical cellular processes such as development, immunological response and cancer have been so far understated.

Despite the findings over the last few years contributed to clarify the relevance of ATRX functions, many issues remain unsolved and compelling research is needed to address critical questions. A number of these questions regards the way by which ATRX suppresses cancer transformation and how mutations in ATRX are so relevant for some specific types of tumors such as adult low-grade glioma and infantile central nervous system tumors. The understanding of the molecular pathways perturbed in ATRX mutated tumors may also open intriguingly therapeutic options such as the employment of ATR inhibitors [86] G4 ligands [139] or PARP1 inhibitors [42] that may exacerbate genomic instability and lead to synthetic lethality.

Another interesting point regards the recently discovered putative RBR RNA binding domain in the ATRX protein that supports the notion of an avid RNA binding protein. Beside the interaction with XIST, TERRA and ChRO1 many other ncRNA may be presumably able to interact with the protein in order to stabilize the binding with other enzymes involved in the regulation of chromatin compaction and gene expression.

In addition, it is well known that mutations in ATRX are necessary but not sufficient for the activation of the ALT telomere maintenance pathway and one of the main unsolved question regards the other molecular actors involved in this mechanism. This aspect is particularly relevant in order to open the possibility to target ALT through pharmacological strategies, inhibiting indefinite replicative potential in this kind of tumors.

At last, an interesting area of research arises from the association of ATRX with the immunological responses. The comprehension of the mechanisms involved in the chromatinization of viral genomes may also be relevant and open questions regarding the presence in the population of ATRX variants that may predispose to or defend from viral infections.

## Figures and Tables

**Table 1:** ATRX interactors, relative binding domains, and function.

PROTEIN	INTERACTION DOMAIN	FUNCTION	REF.
DAXX	DAXX binding domain	H3.3 deposition	[37]
EZH2	EZH2 binding domain	DNA methylation	[38]
HP1α	HP1α binding domain	DNA methylation and PCH formation	[39]
MeCP2	Mecp2 interacting region	DNA methylation and PCH formation	[40]
FANCD2	Unknown	HR	[41]
MRE11	Unknown	DNA DBS Repair, Replicative stress	[42]
NBN	Unknown	DNA DBS Repair, Replicative stress	[43]
RAD50	Unknown	DNA DBS Repair, Replicative stress	[43]
CtIP	Unknown	Replicative stress	[41]
RFC1	Unknown	HR	[44]
PCNA	Unknown	HR	[44]
MacroH2A1.1	322–841 aa	Telomere cohesion	[45,46]
MacroH2A1.2	322–841 aa *	X chromosome inactivation	[45,47]
MacroH2A2	322–841 aa *	X chromosome inactivation	[45]
XIST	RBR domain	X-chromosome inactivation	[48]
TERRA	Probably RBR domain	Telomeric maintenance	[49]
CHRO1	Probably RBR domain	Heterochromatin organization	[50]
H3K9me3	ADD domain	Heterochromatin organization	[51]
H3K4me0	ADD domain	Heterochromatin organization	[52]
SMC1	Unknown	Regulation of mitotic chromosome cohesion	[53]
SMC3	Unknown	Regulation of mitotic chromosome cohesion	[53]
ZNF274	Unknown	Heterochromatin maintenance	[54]
TRIM28	Unknown	Heterochromatin maintenance	[54]
SETDB1	Unknown	Heterochromatin maintenance	[54]

* This interaction domain is referred to MacroH2A1.1 as shown in [46] and it is conceivable to be the same for MacroH2A1.2 and MacroH2A2.

## Data Availability

Not Applicable.
